# Temperature lapse rate estimation and snowmelt runoff simulation in a high-altitude basin

**DOI:** 10.1038/s41598-022-18047-5

**Published:** 2022-08-10

**Authors:** Keke Zhao, Dingzhi Peng, Yu Gu, Xiaoyu Luo, Bo Pang, Zhongfan Zhu

**Affiliations:** College of Water Sciences, Beijing Normal University, Beijing, China

**Keywords:** Climate sciences, Hydrology

## Abstract

As a key parameter of hydrological process modeling, the near-surface air temperature lapse rate reflects the vertical changes in air temperature characteristics in alpine basins but often lacks the support of sufficient ground observation data. This study estimated the lapse rate of the Lhasa River Basin (LRB) from the monthly air temperature dataset (2001–2015), which was derived based on good relationships between the observed air temperature at eight gauged stations and the corresponding gridded land surface temperature of MODIS. The estimated annual average air temperature lapse rate was approximately 0.62 °C/100 m. The monthly lapse rate in different years varied seasonally in the range of 0.45–0.8 °C/100 m; the maximum was in May, and the relatively low value occurred from September to January. The snow cover in the zones with relatively low altitudes showed seasonal variation, which was consistent with the air temperature variation. Permanent snow cover appeared in the area above 5000 m and expanded with increasing elevation.

## Introduction

The air temperature in the alpine basin changes rapidly with elevation, which could lead to most land surface processes presenting significant changes with elevation gradients. For example, vegetation showed sharp transitions, and the land surface rapidly changed from vegetation or soil to snow or ice^1,2^. The air temperature lapse rate is a quantitative indicator used to describe the declining trend of temperature with increasing elevation in alpine basins. The value range of 0.55–0.65 °C/100 m has been widely used for the lapse rate^3^; however, the lapse rate changes with seasonal variation and dry and humidity conditions^4^. Due to the sparse and low-elevation meteorological (MET) stations in mountain regions, the air temperature record from MET stations measured at 2 m above the ground always unable to meet the research needs of lapse rate in mountain regions. With the development of satellite technology, a large amount of high-resolution thermal infrared (TIR) data was used to produce atmosphere, land, and ocean products, the land surface temperature (LSTemp) is one of the land products which retrieved from the TIR data^5^. The LSTemp measured by satellites are the land skin temperature including the uppermost parts of e.g. trees, buildings. The strong relationship between the air temperature and the LSTemp was often used as a common pattern to calibrate air temperature^6–9^. From the view of satellite remote sensing, the land surface is the top layer of the interface between the lower boundary of the atmosphere and the solid earth. In the thermal infrared region, this top layer is a few millimeters thick. The TIR signature received by satellite sensors is determined by surface temperature, surface emissivity/reflectivity, atmospheric emission, absorption and scattering actions upon thermal radiation from the surface, and the solar radiation in daytime^10,11^. In recent decades, many methods have been developed to derive LSTemp^5,12^ from the combination thermal infrared (TIR) signal of sensors, including the mono-window algorithm^13^, the split window algorithm^10^, the day/night LST algorithm^11^, the single channel algorithm^14^ and so on. The Landsat ETM+, MODIS, ASTER and AVHRR, etc., could provide some high-resolution LSTemp products^15^.

**Figure 1:**
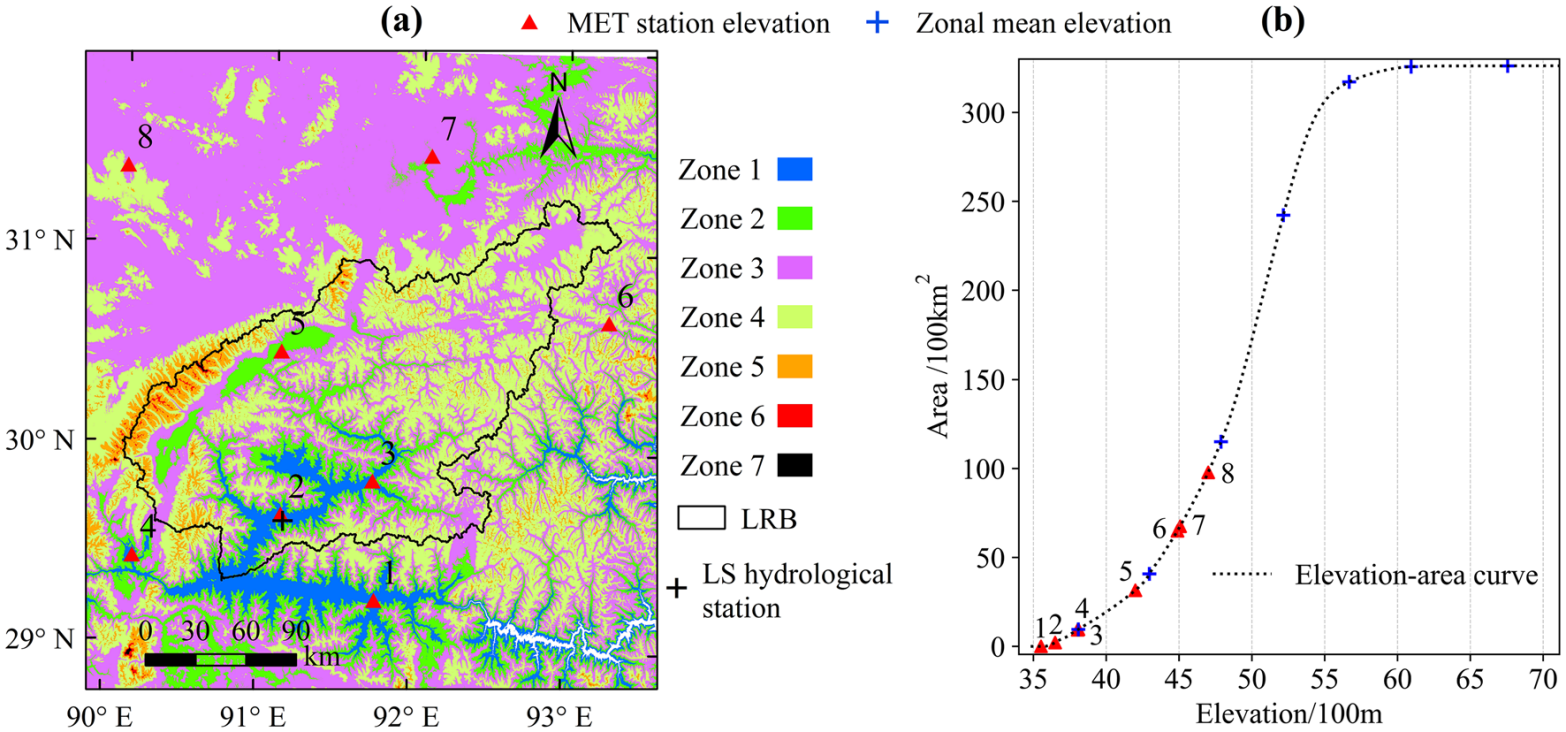
The elevation zones and stations in the LRB. The basin was divided into seven elevation zones at 500 m intervals (Details in Table 1). Based on the 30 m DEM, the elevation-area curve of the LRB was plotted in (**b**), and the mean elevation of each elevation zone is marked on the curve. Eight MET stations were also marked on the curve according to their elevation. In (**a**) was generated with ArcGIS 10.6 (https://www.esri.com/en-us/home).

The surface temperature has a main influence on the hydrological cycle, particularly in the mountain cryosphere, where the water supply is dominated by melting snow or ice^16–19^. For glacier and snow melting runoff modeling, the lapse rate is the key parameter, which would have a direct impact on the simulated accuracy of hydrological processes in ungauged basins^20–22^. The permanent snow and ice cover were likely to be strongly reduced or even eliminated with rising air temperatures^23^. Long-term snow cover across the Tibetan Plateau was investigated, and a significant decreasing trend in the duration of snow cover was found to be consistent with climate change^24^. By analyzing remote sensing snow cover products of the Tibetan Plateau, the snow-covered area shows a mean decrease over the entire plateau but presents completely different trends in low and high elevation ranges^25^. The length of the snow-free season increased at lower-elevation sites, as opposed to decreasing at higher elevations in eastern Tibet^19^. The Lhasa River (Fig. 1a) Basin (LRB), a subbasin in the Tibetan Plateau, is a typical alpine region with large vertical differences over relatively short horizontal distances (3481–7112 m). More researches have focused on simulating hydrological progress^26,27^, and the research field of the changing process of air temperature and snow cover with increasing elevation in the LRB has been less studied. Sparse and inhomogeneous gauge-based records bring great uncertainty for studying the spatiotemporal distribution and changing rules of the air temperature and bring much uncertainty to simulate the hydrological progress.

**Table 1:** Detail information of the elevation zones and MET stations in the LRB.

No	Station (Abbreviation)	Elevation/m	Elevation zone	Elevation range/m	Area ratio/%
1	Zedang (ZD)	3552	Zone 1	3481–4000	5.94
2	Lhasa (LS)	3649			
3	Mozhugongka (MZGK)	3804			
4	Nimu (NM)	3809			
5	Dangxiong (DX)	4200	Zone 2	4000–4500	14.46
6	Jiali (JL)	4489			
7	Naqu (NQ)	4507	Zone 3	4500–5000	32.37
8	Bange (BG)	4700			
–	–	–	Zone 4	5000–5500	41.12
–	–	–	Zone 5	5500–6000	5.74
–	–	–	Zone 6	6000–6500	0.35
–	–	–	Zone 7	6500–7112	0.02

The purpose of this study is to address two topics. First, we estimated the air temperature lapse rate and analyzed the distribution features of air temperature in the LRB based on a reconstructed air temperature product obtained from the global MODIS LSTemp product and collected gauge-based air temperature. Second, the spatial–temporal snow cover patterns across the LRB are quantified by using remote sensing data. Furthermore, the rationality and accuracy of the air temperature reconstructions are verified by the variation features of snow cover and a snowmelt runoff model (SRM)^28^ simulated with the main variables of air temperature, precipitation, snow cover and temperature lapse rate.

## Results and discussion

### Relationship between air temperature and LSTemp

The MOD11C3 LST product (https://doi.org/10.5067/MODIS/MOD11C3.061) is derived by reprojection, composite, average and aggregate of two MODIS LST products at $0.05^\circ$ grids. These two component products are retrieved from the data in MODIS TIR bands by using the day/night LST algorithm and the generalized split-window, respectively.

**Figure 2:**
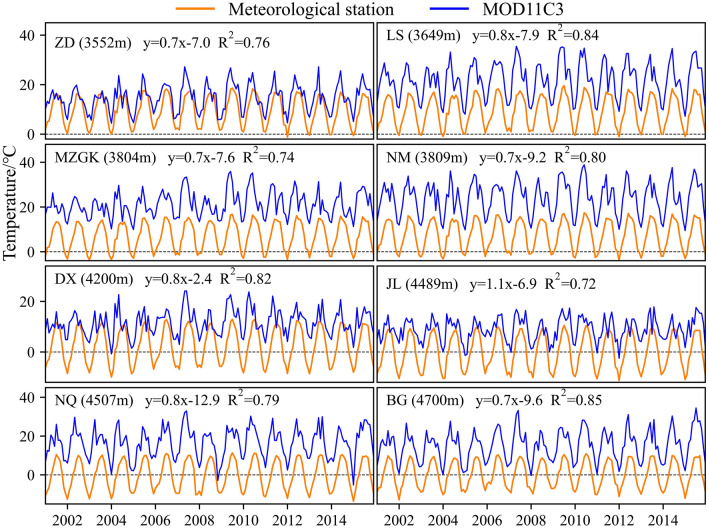
Comparison of air temperature of eight MET stations with corresponding gridded LSTemp of MOD11C3 from 2001 to 2015. Taking air temperature as y and LSTemp as x, the linear equation for each combination was fitted and marked as text in the subgraph.

**Figure 3:**
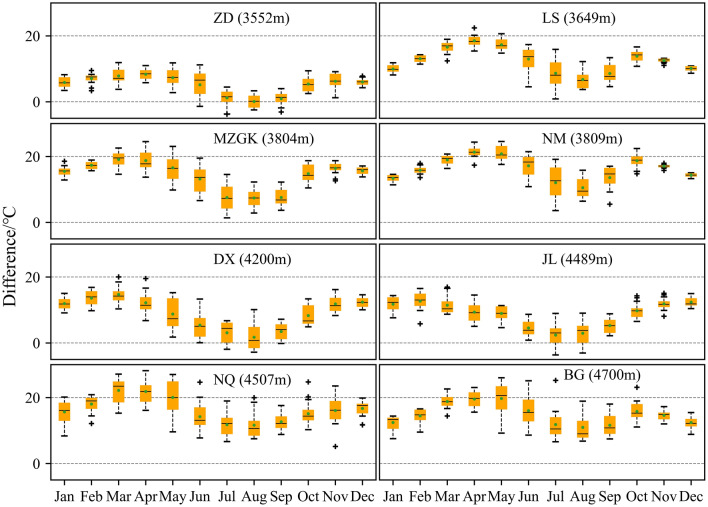
Boxplots of the D-value between LSTemp and air temperature. All eight diagrams had the same X- and y-axis scales and each boxplot were marked with 15 different values of 2001–2015.

The large area above 4700 m of the LRB (approximately 70% area) lacks the air temperature record of the ground station, there are three meteorological stations within the basin and five stations around the basin (Table 1 and Fig. 1a). Figure 2 show good linear relationship of air temperature with the corresponding gridded LSTemp. The time series of these two kinds of temperature all change with season, highest in summer (around June to August, warm season) and lowest in winter (around November to February of the next year, cold season). LSTemp is generally higher than air temperature in the whole year of 2001–2015 (Fig. 2). The difference value plots of Fig. 3 show that a small amount of the negative values appears in June to September for ZD, DX and JL, and the rest are all positive. Overall, the difference values of ZD, DX and JL were relatively low, and the largest values were all less than 20 °C. For LS, MZGK, NM, NQ and BG, the smallest difference values were all above 0 °C, and the largest values were all above 20 °C. Combined with the linear equation between air temperature (y) and LSTemp (x), all linear coefficients except JL are less than 1 (approximately 0.7 and 0.8), and all intercept values are negative. Taking full advantage of limited records of MET stations appears to confirm that the LSTemp of MOD11C3 is higher than the corresponding air temperature, and the difference between the two is obviously increased in the cold season than in the warm season.

Relevant research reveals that the relationship between LSTemp and observed air temperature at 2 m above the ground is characterized with respect to land cover and elevation^7^. With increasing solar radiation and air temperature, the specific heat capacity of snow and the freezing point are reached, after which the snow begins to melt rapidly. Snow surfaces have the highest albedos in nature and the surface albedo is very sensitive to changes in snow cover area^29^. The snow albedo effect directly impacts the shortwave radiation absorbed at the surface and subsequently alters turbulent fluxes, resulting in changes in surface air temperatures via diabatic heating/cooling^29,30^, therefore, there is no doubt that the feedback of snow on air temperature in land-surface-atmosphere interaction system changes with the variation of snow cover area in different seasons^31^. That is also an explanation for why the difference between the air temperature and the LSTemp varies with season.

### Estimation of gridded air temperature

**Figure 4:**
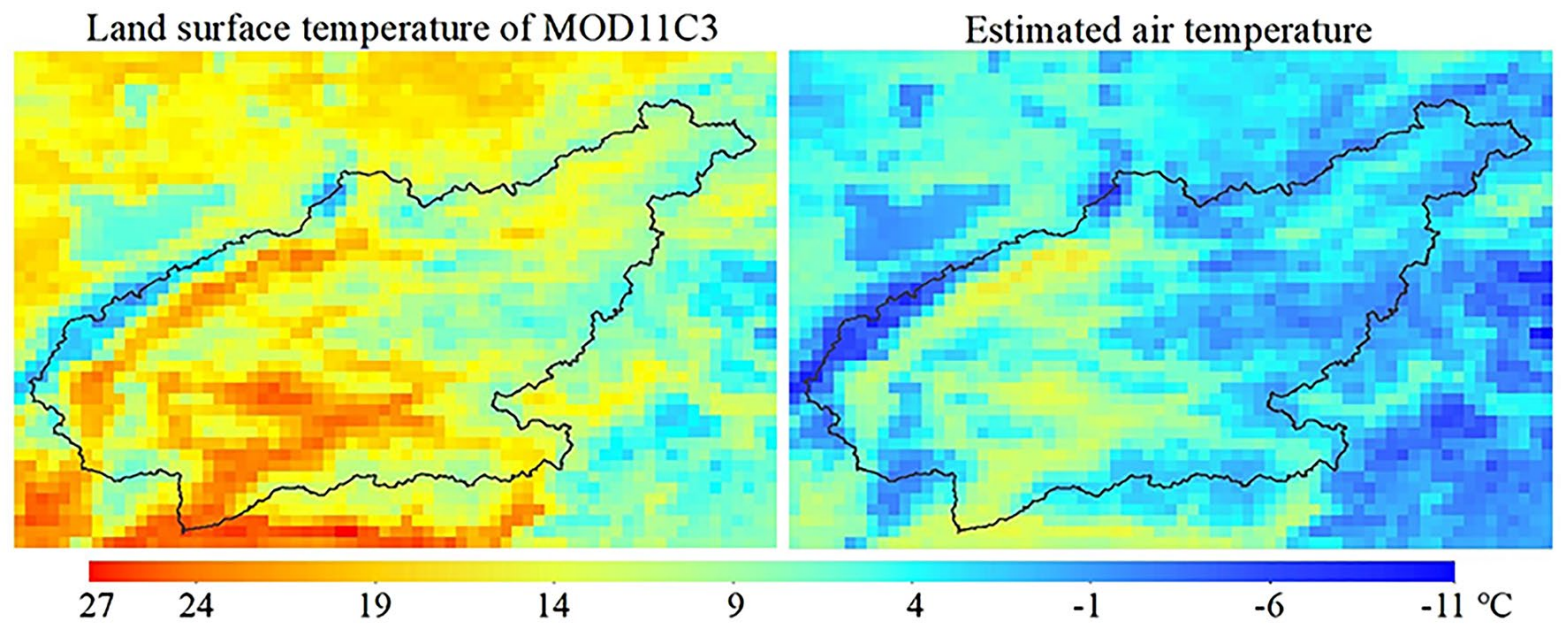
Distribution maps of monthly average temperature between 2001 and 2015 with 0.05° resolution.

Based on the analysis of a good linear relationship between station air temperature and gridded LSTemp, an inverse distance weighted (IDW) method was used to interpolate local linear equation parameters (coefficient, intercept and residual) globally. Then, the three interpolation datasets of coefficient, intercept and residual were combined with MOD11C3 as the input data to calculate the air temperature in the $0.05^\circ$ grid. We display the spatial distribution comparison of monthly average temperature (2001–2015) of LSTemp and air temperature in a raster map (Fig. 4). It is clear that the numerical regional characteristics of the two maps are all consistent with the spatial distribution of elevation zones in Fig. 1a; the monthly average range of MOD11C3 and air temperature are from 4 to 27 °C and from − 11 to 13 °C, respectively, and MOD11C3 is significantly higher than the estimated air temperature at the same row and column position. Further statistical results show that the monthly average temperatures of the two maps for the whole basin are 11.8 °C and 2.2 °C.

### Analysis of temperature lapse rate

**Figure 5:**
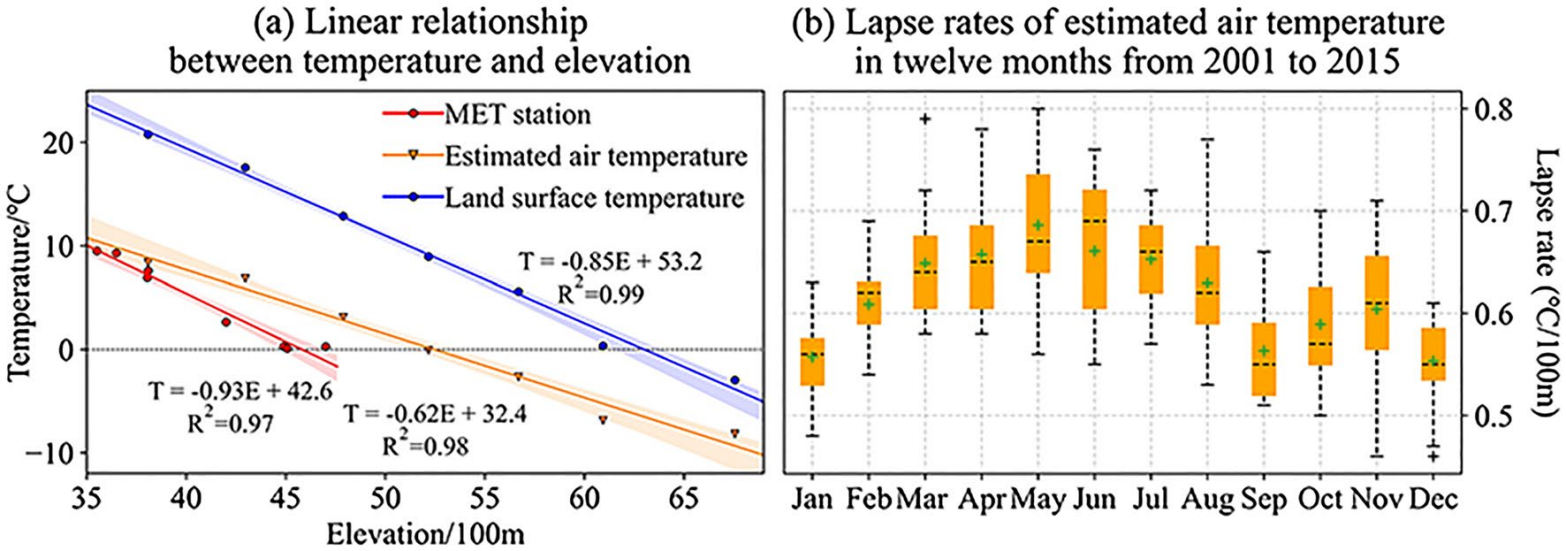
Relationship between elevation and different temperature datasets and variation of air temperature lapse rate in different months. In (**b)**, the boxplot of each month is composed of 15 temperature lapse rates from 2001 to 2015, which were obtained by constructing the trend line between the zonal average temperature and zonal average elevation of 7 elevation zones.

The temperature lapse rate is the gradient value of the trend line between temperature and elevation. The trend line of observed air temperature is fitted by the monthly average temperature and the elevations of eight MET stations. For two gridded datasets, the construction of trend lines was based on the zonal average temperature and zonal average elevation of 7 elevation zones (Fig. 1a). The results in Fig. 5a show that temperature decreases with elevation in a good linear relationship because all fitting degrees (R^2^) are extremely close to 1, but there are numerical differences for the three temperature lapse rates obtained from different data sources. The observed air temperature decreases by 0.93 °C with elevation increases of 100 m in the elevation range of 3552–4700 m. For the estimated gridded air temperature and the LSTemp distributed in the whole basin, the global lapse rates are 0.62 °C/100 m and 0.85 °C/100 m, respectively. That is, not only is the LSTemp higher than the near-surface temperature, but the lapse rate of the land surface is also larger than that of the near-surface. We may conclude that the gap between LSTemp and near-surface temperature reduces gradually with increasing elevation. The observed air temperature lapse rate of 0.93 °C/100 m lacks representation because it is calculated from the local scattered point temperature records, and there are five MET stations we used that are located outside the basin. However, the high R^2^ (0.97) of the trend line reflects that a good fitting relationship can still be obtained between elevation and air temperature when other factors are ignored. It also reflects from the side that elevation is a key factor for temperature changes.

Based on the dataset of estimated air temperature, the global temperature lapse rates of all months from 2001 to 2015 are calculated and compared with the format of the boxplot (Fig. 5b). The lapse rate values are distributed in the range of 0.45–0.8 °C/100 m, and the median and mean for different month boxplots vary to some degree. Overall, the maximum appears around May, and the relatively low values are concentrated from September to January. Combined with the features of seasonal temperature change, it is concluded that the rise in air temperature is accompanied by a rise in the lapse rate and vice versa.

### Variations in snow cover

**Figure 6:**
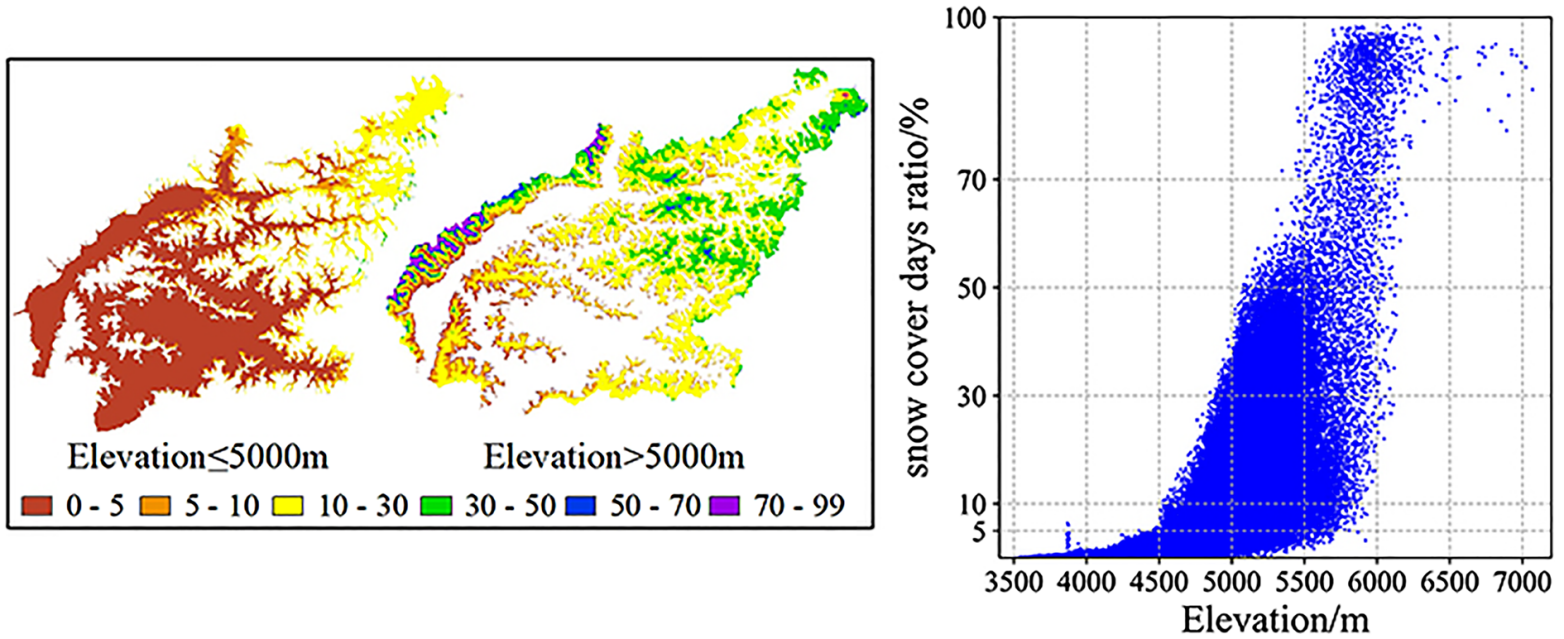
Snow cover days ratio (%). The daily MODIS cloud-free snow cover product over the Tibetan Plateau (2002–2015) with 0.005° resolution^32^ was used to analyze the distribution characteristics of snow cover. The left figure showed the ratio of the proportion of days covered by snow to the total days from 2003 to 2014. To obtain intuitive changes in snow cover days with elevation, the gridded result is divided into two maps according to the boundary elevation of 5000 m. The right scatter point is about the snow cover days ratio and elevation of all grids at a 0.005° resolution. The left figure was generated with ArcGIS 10.6 (https://www.esri.com/en-us/home).

Regardless of time or space, temperature is the decisive factor for the distribution of snow cover. In view of the linear relationship between temperature and elevation, here, high-precision DEM data with a 30-m resolution are used to analyze the snow cover. Figure 6 shows the difference in snow cover days in the whole basin. Below the elevation of 5000 m (zones 1, 2 and 3), the snow cover days ratio concentrates in the range of 0–5%, and with increasing elevation, the days ratio can reach 10–35%. Further with elevations above 5000 m up to 7112 m (zones 4, 5, 6 and 7), the ratio also gradually increases to 99%. Statistics show that the ratio in the range of 0–5% accounts for 41% of the LRB, followed by the range of 10–30%, 5–10% and 30–50%, accounting for 33%, 14% and 10% of the total area, respectively. The remaining 2% of the area (snow cover days ratio > 50%) contains a part of elevation zones 4, 5 and 6 and the whole area of zone 7, where more than half of the time there is snow coverage.

**Figure 7:**
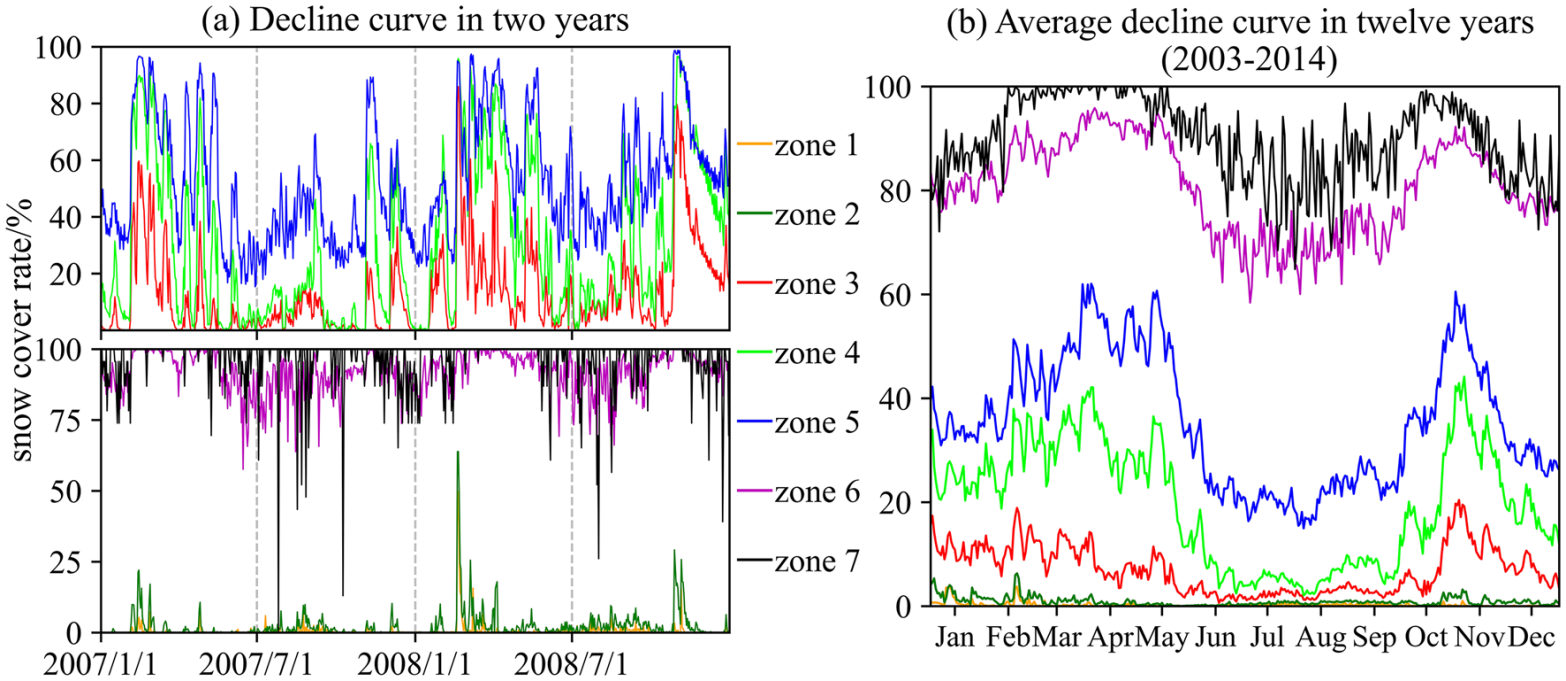
Decline curves of elevation zones. Taking each elevation zone as a unit, the decline curves are made of the daily ratio that snow cover area accounts for the elevation zone area. In (**a**) shows an example of the snow cover area ratio variation on a daily scale between January 1, 2007, and December 31, 2008. By ignoring the ratio of the last day of the intercalary month between January 1, 2003, and December 31, 2014, the average decline curve is computed based on the decline curves of 12 different years for each elevation zone, as shown in (**b**).

It is obviously shown in Fig. 7 that the higher the elevation zone, the larger the snow cover rate. For elevation zones 1 and 2, there are no large ups and downs for the decline curves, and the values of the whole year are almost 0; new snow in the cold season melts in a short time. The high oscillation frequency of the daily decline curves presents the sensitivity of snow cover to temperature change. In the elevation area above 4500 m, a large proportion of snow melted from June to September, and the snow cover increased from October to May. Similar to the largest zone of elevation 4, the snow cover rate could reach approximately 90% in the cold season and then drop to almost 0% but never equal 0% in the warm season for each year. Combined with the observation that a snow cover days ratio above 50% begins to appear in elevation zone 4, it is reasonable to conclude that permanent snow cover begins to appear in this zone, and as the elevation continues to rise, the area of permanent snow gradually expands in elevation zones 5, 6, and 7. As shown in Fig. 5a, the zonal average monthly air temperature starts to less than 0 °C from zone 4 and reaches a lower value with a linear decline trend of 0.62 °C/100 m, which can well explain the existence and expansion of permanent snow and the high snow cover rate for the zones above 5000 m.

### Simulation of snowmelt runoff

**Figure 8:**
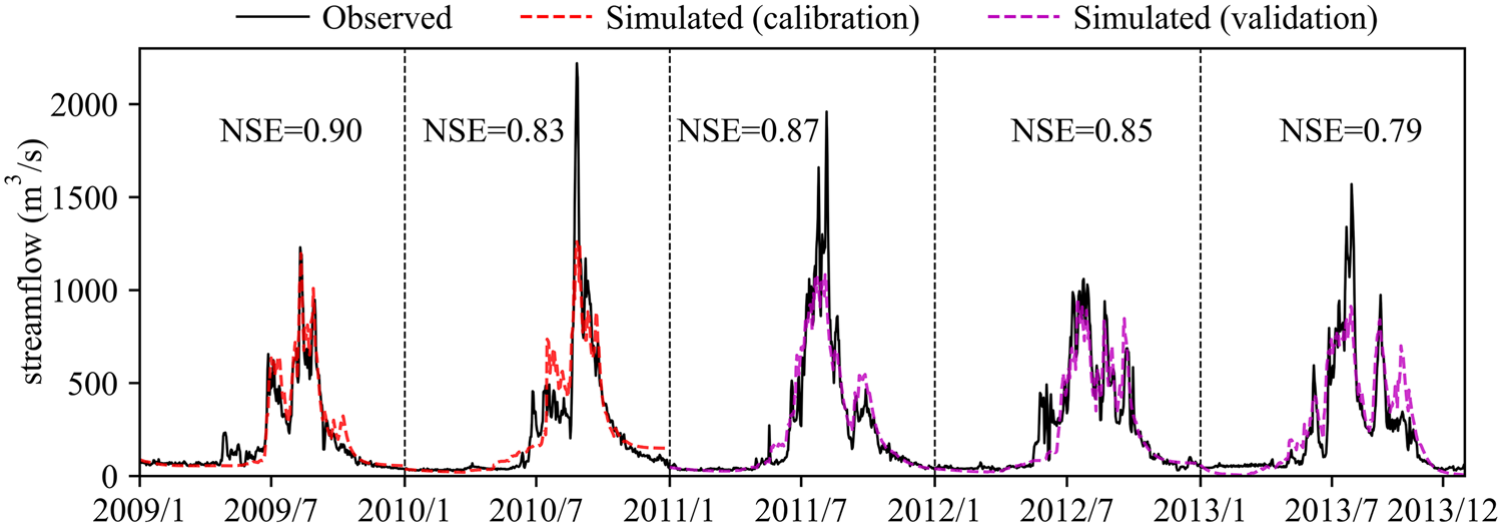
Results of SRM. In the calibration (2009–2010), parameters including the degree day factor (${\upalpha } = 0.13\;{\text{cm}}\;^{ \circ } {\text{C}}^{ - 1} \;{\text{day}}^{ - 1}$), critical temperature (0 °C), runoff coefficient for snow ($C_{S} = 0.3$) and runoff coefficient of rain ($C_{R} = 0.5$) were determined, and NSE reached 0.9 and 0.83, respectively. These four parameters were used in the validation (2011–2013), and NSE were 0.87, 0.85 and 0.79, respectively.

Although SRM is a simple conceptual and degree-day model, it is widely used to simulate the snowmelt runoff in numerous mountain basins of the world^33^. And the simulation periods of SRM can be a week, a snowmelt season, a year, or a sequence of years. According to the Formula (7), the SRM was constructed in LRB based on seven elevation zones that were delineated in intervals of approximately 500 m (Fig. 1a), and calculated with three main input variables: (1) the daily air temperature of average hypsometric elevation in each zone, which was extrapolated by the daily reference record of the LS MET station and the monthly lapse rate in Fig. 5b, (2) the decline curves of snow cover in seven zones, (3) and the daily average precipitation of each zone, which was extracted from the GPM IMERG Final Run product (download the product via: https://gpm.nasa.gov/data/directory). The daily observed streamflow of the LS hydrological station (Fig. 1a) was used as the reference streamflow, and the Nash–Sutcliffe efficiency (NSE) was adopted as the evaluation index of the SRM. The simulation results in Fig. 8 show that the input data we produced can well simulate the process of streamflow in both the calibration years of 2009 (NSE = 0.9) and 2010 (NSE = 0.83), and the validation years of 2011, 2012 and 2013 (NSE are 0.87, 0.85 and 0.79, respectively).

However, it is obvious in Fig. 8 that there is a large gap between the observed sharp peaks that occurred in approximately August 2010, 2011 and 2013 and the corresponding relatively low simulated streamflow, although the NSE values are high for 2010 and 2011. In such cases, SRM designers explain^33^ that sharp peaks are typical for heavy rainfall runoff, which is often concentrated in a short time interval, as opposed to the relatively regular daily fluctuations for snowmelt runoff. Adjustment of the rainfall threshold and parameter of recession coefficients x and y for sharp runoff peaks from occasional heavy rainfalls are necessary, but the final results of 2010, 2011 and 2013 reveal that the effort for raising the simulated streamflow peaks is weak. Actually, frequent occurred rain-on-snow events in high-elevation areas was proved to be responsible for flood generation^34,35^, and 21% and 70% of the peak flows were associated with rain-on-snow event in parts of Bavaria^36^ and Austria^37^, respectively. The SRM without considering the runoff contribution of rain-on-snow events may underestimate the peak flow.

## Conclusion

In alpine basins with large elevation differences, the average air temperature decreases with a linear trend from low altitude to high altitude. This study reconstructed the high space resolution air temperature by analyzing the relationship between gauge-based air temperature and MODIS LSTemp and estimated the monthly air temperature lapse rate of the LRB. Combined with the variation in snow cover in time and space, which is directly affected by air temperature changes, the precision of the air temperature reconstructions was verified. In view of the calculation of SRM heavily relying on the inputs and parameters of air temperature, the depletion curves of snow cover and the lapse rate, the well simulation results of the SRM model further confirm the pattern we concluded, and detailed achievements are as follows:

1. MODIS LSTemp is basically larger than gauge-based air temperature, and the difference presents significant seasonal variations. The smallest occurs in summer (around July to Sep), and the largest occurs in spring and autumn (around Apr and Oct). The good correlation relationship of the two temperatures provides a basis for reconstructing air temperature.
2. The annual average air temperature lapse rate is 0.62 $\mathrm{^\circ{\rm C} }/100\mathrm{ m}$, which is less than the LSTemp of 0.85 $\mathrm{^\circ{\rm C} }/100\mathrm{ m}$. The air temperature lapse rate varies in the range of 0.45–0.8 $\mathrm{^\circ{\rm C} }/100\mathrm{ m}$ for different months. The largest lapse rate occurred in approximately May, and the relatively low values were concentrated from September to January.
3. Spatiotemporal variations in snow coverage measured by remote sensing are related to the reconstructed air temperature. The annual average air temperature starts to less than $0 ^\circ \mathrm{C}$ in the area above an elevation of approximately 5000 m and continues to decrease with increasing elevation. Permanent snow coverage also appears and expands with subzero air temperatures. For the area below 5000 m, snow cover displays seasonal variation with the features of accumulation in the cold season and almost all melting in the warm season.
4. The daily air temperature of each elevation zone calculated by our estimated monthly lapse rate and the decline curves of each zone used as the main input variables can simulate well-fitted streamflow by using the SRM model.

## Methods

### Construction of air temperature

To explore the relationship between gauge-based air temperature and corresponding gridded MODIS LSTemp, the statistical regression method was the first choice to describe the possible relation. Assuming that the MODIS LSTemp is *y′*, the observed air temperature is *y*. The structure of the linear regression model is as follows:

1 $$y_{\alpha } = \beta_{1} y_{\alpha }^{\prime } + \beta_{0} + \varepsilon_{\alpha }$$

where $\beta_{1}$ is the linear regression coefficient; ${ }\beta_{0}$ is the intercept; $\varepsilon_{\alpha }$ is the residual error; and $\alpha$ refers to the meteorological station.

The inverse distance weight (IDW) interpolation method^38,39^ is adopted to calculate global parameters. An important assumption of IDW is that the surface stations have great effects on local interpolation points, and the degree of influence on interpolation points decreases with increasing distance. The value of an interpolation point would be affected by the nearest *N* surface stations, and there is an inverse proportional relationship between the degree of influence and distance; surface stations close to the interpolation point have a large weight. The mathematical expression of the inverse distance weight method is as follows:

2 $$\hat{Z}_{0} = \mathop \sum \limits_{i = 0}^{{\text{n}}} \left( {Z_{i} ,Q_{i} } \right)$$

3 $$Q_{i} = \frac{{f\left( {d_{j} } \right)}}{{\mathop \sum \nolimits_{j = 1}^{n} f\left( {d_{j} } \right)}}$$

4 $$f\left( {d_{j} } \right) = \frac{1}{{d_{j}^{b} }}$$

where $\hat{Z}_{0}$ is the estimated value at the point of interpolation (*x*_*0*_, *y*_*0*_); $Z_{i}$ is the value at the point of observation (*x*_*i*_, *y*_*i*_); $Q_{i}$ is the weight coefficient between interpolation point (*x*_*0*_, *y*_*0*_) and observation point (*x*_*i*_, *y*_*i*_); $f\left( {d_{j} } \right)$ is the weight function of distance $d_{j}$; when *b* = 1, the method is inverse distance reciprocal interpolation; when *b* = 2, the method is inverse distance reciprocal square interpolation; and *n* is the number of observation points involved in the calculation.

As a global interpolation algorithm, all discrete observation points participate in the estimation of each value of the interpolation point. It combines the advantages of the natural neighborhood method of the Thiessen polygon and the multiple regression gradient method, considers the distance factor, allocates the weight for the discrete observation points according to the distance, and when anisotropy occurs, the weight of the direction is also considered. With increasing distance between the observation point and the interpolation point, the weight shows a decreasing trend of power function. However, the traditional IDW method is particularly sensitive to the selection of the weight function. There are subtle differences in the weight function, which will cause large fluctuations in the generated results, and it is easily affected by the observation point dataset. When some data at observation points are significantly higher or lower than the average level, there will be an isolated distribution mode and a large deviation in local interpolation.

### Snow cover rate and snow cover days ratio

When the temperature is lower than the critical temperature $\left( {T_{CRIT} } \right)$, the precipitation event will be treated as new snow, and the snow cover depth and area can be expected to increase; this is the so-called snow accumulation period. In contrast, when the temperature is higher than $T_{CRIT}$, snowpack starts to melt, and melting accelerates with increasing temperature, which is the so-called snowmelt period. In high alpine basins, changing characteristics of snow cover area and days can reflect the temperature variation. This paper adopted the depletion curves of the snow coverage and snow cover days ratio to present the seasonal variations and vertical distribution of snow cover. Based on the 0.005° × 0.005° resolution product of MODIS daily cloud-free snow cover, the depletion curves of the snow coverage in seven elevation zones of the LRB are composed and drawn by the daily snow cover rate:

5 $$snow \,\,cover\,\, rate = \frac{snow\,\, cover \,\,area}{{total \,\,area\,\, of \,\,elevation \,\,zone}} \times 100\%$$

Counting days covered with snow of each grid in 2002–2015, the formula for the ratio of days covered by snow is as follows:

6 $${ }snow \,\,cover\,\, days \,\,ratio = \frac{Days \,\,of \,\,snow \,\,cover}{{total \,\,number\,\, of \,\,days}} \times 100{{\% }}$$

### Snowmelt runoff model

The SRM^28^ is designed for mountain regions with large elevation difference to compute the streamflow, which is composed of snowmelt and rainfall of different elevation zones. The degree-day method in SRM is reliable for computing total snowmelt depths for periods of a week to the entire snowmelt season. Daily average air temperature, daily average precipitation and daily snow cover rate of each elevation zone are basic input variables, the degree-day factor $\left( {{\upalpha },{\text{ cm}}\;^{ \circ } {\text{C}}^{ - 1} \;{\text{day}}^{ - 1} } \right)$, the temperature lapse rate $\left( {\gamma , \;^{ \circ } {\text{C/}}100\;{\text{m}}} \right)$, the critical temperature $\left( {T_{CRIT} ,^{ \circ } {\text{C}}} \right)$, the runoff coefficient to snowmelt $\left( {C_{S} } \right)$, the runoff coefficient to rain $\left( {C_{R} } \right)$, the recession coefficient *x* and *y* are the necessary parameters. Here, the key parameter degree-day factor ${\upalpha }$ indicates the snowmelt depth caused by 1 °C increasing in one day (1 degree-day)^28^. In the defined LRB with seven elevation zones, construction of SRM is shown as formula (7). The simulated streamflow on day $n + 1$ is formed from the contribution of snowmelt, precipitation and the discharge on day $n$, and the contribution proportion is determined from the recession coefficient $k_{n + 1}$ which is adjusted with constant *x* and *y* in the calibration (Formula (8)).

7 $$Q_{n + 1} =\left\{ \begin{gathered} \left[ {C_{S} \cdot \alpha \left( {T_{n} + {\Delta }T_{n} } \right)S_{1n} + C_{R} P_{1n} } \right]\frac{{A_{1} \cdot 10000}}{86400} + \hfill \\ \left[ {C_{S} \cdot \alpha \left( {T_{n} + {\Delta }T_{n} } \right)S_{2n} + C_{R} P_{2n} } \right]\frac{{A_{2} \cdot 10000}}{86400} + \hfill \\ \left[ {C_{S} \cdot \alpha \left( {T_{n} + {\Delta }T_{n} } \right)S_{3n} + C_{R} P_{3n} } \right]\frac{{A_{3} \cdot 10000}}{86400} + \hfill \\ \left[ {C_{S} \cdot \alpha \left( {T_{n} + {\Delta }T_{n} } \right)S_{4n} + C_{R} P_{4n} } \right]\frac{{A_{4} \cdot 10000}}{86400} + \hfill \\ \left[ {C_{S} \cdot \alpha \left( {T_{n} + {\Delta }T_{n} } \right)S_{5n} + C_{R} P_{5n} } \right]\frac{{A_{5} \cdot 10000}}{86400} + \hfill \\ \left[ {C_{S} \cdot \alpha \left( {T_{n} + {\Delta }T_{n} } \right)S_{6n} + C_{R} P_{6n} } \right]\frac{{A_{6} \cdot 10000}}{86400} + \hfill \\ \left[ {C_{S} \cdot \alpha \left( {T_{n} + {\Delta }T_{n} } \right)S_{7n} + C_{R} P_{7n} } \right]\frac{{A_{7} \cdot 10000}}{86400} \hfill \\ \end{gathered} \right\}\left( {1 - k_{n + 1} } \right) + Q_{n} k_{n + 1}$$

8 $$k_{n + 1} = x \cdot Q_{n}^{ - y}$$

where $T$ is the number of degree-days $\left( {^{ \circ } {\text{C}}\;{\text{ day}}} \right)$ which determined from the difference value between air temperature and an adopted reference temperature (melting point of snow); ${\Delta }T$ is the adjustment value by the temperature lapse rate $\left( {^{ \circ } {\text{C}}\;{\text{ day}}} \right)$; $S_{1}$, $S_{2}$,…, $S_{7}$ are the snow cover rate of each elevation zone $\left( {\text{\% }} \right)$, respectively; $P_{1}$, $P_{2}$,…, $P_{7}$ are the average precipitation of each elevation zone $\left( {{\text{cm}}} \right)$, respectively; $A_{1}$, $A_{2}$,…, $A_{7}$ are the area of each elevation zone $({\text{km}}^{2} )$, respectively.

Snow as the fundamental source of solid water, its area shows the linear relationship with the amount of meltwater supply for mountain regions. The larger the snow cover area, the more snow meltwater in the simulation of SRM. In the melting period, the snowmelt depth of each zone (calculated by the degree-day factor ${\upalpha }$ and the zonal degree-days $T_{n} + {\Delta }T_{n}$) (cm/day) is transformed to snowmelt volume (cm km^2^/day) by multiply the snow cover area, and further transformed to standard unit of streamflow ${\text{(m}}^{{3}} {\text{/s)}}$ by multiply the 10,000/86,400. In the accumulation period, air temperature together with $T_{CRIT}$ determine whether the precipitation immediately contributes to streamflow (rain) or snowfall take place.

Previous study showed that the sensitivity of SRM parameters ranked as follows: ${\upalpha } > {\upgamma } > C_{S} > C_{R} > T_{CRIT}$, and successfully simulated the snowmelt runoff process of 2002 and 2003 in LRB^26^.

## Supplementary Information

Supplementary Information 1.

Supplementary Information 2.

Supplementary Information 3.

## Data Availability

MOD11C3 LST, MODIS daily cloud-free snow cover in the Tibetan Plateau (2002–2015) and GPM IMERG were downloaded from https://doi.org/10.5067/MODIS/MOD11C3.061, http://www.tpdc.ac.cn/ and https://gpm.nasa.gov/data/directory, respectively. The air temperature in the meteorological stations was downloaded from http://data.cma.cn. The estimated air temperature dataset, the data of snow cover days ratio with elevation, and the observed air temperature of four meteorological stations with corresponding MOD11C3 LST could be obtained from the supplementary files.
